# Radiation Dose-Rate: Engelward and Yanch Respond

**DOI:** 10.1289/ehp.1205595R

**Published:** 2012-11-01

**Authors:** Bevin Engelward, Jacquelyn Yanch

**Affiliations:** 1 Department of Biological Engineering, Massachusetts Institute of Technology, Cambridge, Massachusetts, E-mail: bevin@mit.edu; 2 Department of Nuclear Science and Engineering, Massachusetts Institute of Technology Cambridge, Massachusetts

Melzer raises many interesting points regarding our study of low dose-rate radiation (Olipitz et al. 2012). Responding to his letter gives us the opportunity to clarify the rationale behind some of our approaches and interpretations.

Melzer points out that sample sizes in our study varied from 6 to 60. This is absolutely true because it was necessary to adjust sample sizes according to the end point being analyzed. Larger cohorts are required under conditions where there is higher variance, which is the case for the FYDR (fluorescent yellow direct repeat) mice. Smaller cohorts are sufficient when the variance is lower, such as for micronuclei.

Melzer notes that we used transgenic mice for one end point and normal mice for others. In our study, all of the animals were isogenic (C57Bl6), with the only difference being the insertion of the reporter transgene into the FYDR mice. We have not observed any biological impact of this insertion, and the insertion was made in only one of the two copies of chromosome 1, making it even less likely to affect the biology of the animal. Even if there were an impact, this would not compromise the approach because each end point of the study is appropriately internally controlled. Because each end point was evaluated relative to an isogenic control cohort, the approach did not weaken the ability to detect effects but actually strengthened the method.

In his letter, Melzer correctly points out that data of Tanaka et al. (2009) show a statistically significant increase in chromosome aberrations in cells from mice exposed to 1 mGy/day up to a total of 1,000 mGy. However, after exposure to that same dose-rate for a longer period (up to 8,000 mGy), there was no statistically significant change in the number of chromosome aberrations. Furthermore, Tanaka et al. (2009) stated that

Regression coefficients (b4 and b3) in the equations for Dic by FISH at low dose rates of 20 mGy/day and 1 mGy/day at doses less than 8,000 mGy were not statistically significant.

Tanaka et al. also stated that

It remains to be clarified whether the dose–response relationship for Dic+Rc, UA or Dic by FISH was significantly different for dose-rates of 1 mGy/day and 20 mGy/day or whether the linear dose–response relationship at 1 mGy/day was significantly different from the spontaneous level.

Melzer is correct that we cannot rule out the possibility that genetic changes might have been observed if the exposures had been carried out for a longer period. However, because cells have the capacity to repair radiation-induced DNA damage, it is possible that DNA damage would not accumulate with time.

Melzer is correct that a recent report suggested that exposure to radiation from CT (computed tomography) scans affects the risk of cancer in exposed children. An important difference is that CT scans are an acute exposure at a high dose-rate, which is very different from the low dose-rate conditions in our study. Nevertheless, we agree that it is very important to consider the fact that children have increased sensitivity to radiation damage.

One of Melzer’s final points is that inflammation might affect radiation sensitivity. Although we did not test the impact of inflammation in our study, it is important to note that inflammation is a highly genotoxic process itself, leading to levels of DNA damage orders of magnitude higher than levels we calculated in response to the low dose-rate we used. Finally, Melzer raises the issue of internal exposure by ingestion versus external sources. The body handles ingested radionuclides according to the chemical behavior of the element. Although we have examined the effect of radiation dose as delivered by internal or external photon- or beta-emitters, we did not consider the internal pattern of radionuclide uptake in our study.
